# Plasma membrane-association of SAUL1-type plant U-box armadillo repeat proteins is conserved in land plants

**DOI:** 10.3389/fpls.2014.00037

**Published:** 2014-02-19

**Authors:** Katja Vogelmann, Christa Subert, Nina Danzberger, Gabriele Drechsel, Johannes Bergler, Tanja Kotur, Thorsten Burmester, Stefan Hoth

**Affiliations:** ^1^Molekulare Pflanzenphysiologie, Biozentrum Klein Flottbek, Universität HamburgHamburg, Germany; ^2^Department Biologie, Molekulare Pflanzenphysiologie, Friedrich-Alexander-Universität Erlangen-NürnbergErlangen, Germany; ^3^Zentrum für Molekularbiologie der Pflanzen, Allgemeine Genetik, Universität TübingenTübingen, Germany; ^4^Stoffwechselphysiologie, Biozentrum Grindel, Universität HamburgHamburg, Germany

**Keywords:** SAUL1, U-box, ARM repeat, armadillo, plasma membrane, ubiquitin

## Abstract

Post-translational protein modification plays a pivotal role in the regulation and specific turnover of proteins. One of these important modifications is the ubiquitination of target proteins, which can occur at distinct cellular compartments. At the plasma membrane, ubiquitination regulates the internalization and thus trafficking of membrane proteins such as receptors and channels. The Arabidopsis plant U-box (PUB) armadillo repeat (PUB-ARM) ubiquitin ligase SAUL1 (SENESCENCE-ASSOCIATED UBIQUITIN LIGASE1) is part of the ubiquitination machinery at the plasma membrane. In contrast to most other PUB-ARM proteins, SAUL1 carries additional C-terminal ARM repeats responsible for plasma membrane-association. Here, we demonstrated that the C-terminal ARM repeat domain is also essential and sufficient to mediate plasma membrane-association of the closest Arabidopis paralog AtPUB43. We investigated targeting of PUB-ARM ubiquitin ligases of different plant species to find out whether plasma membrane-association of SAUL1-type PUB-ARM proteins is conserved. Phylogenetic analysis identified orthologs of SAUL1 in these plant species. Intracellular localization of transiently expressed GFP fusion proteins revealed that indeed plasma membrane-association due to additional C-terminal ARM repeats represents a conserved feature of SAUL1-type proteins. Analyses of transgenic Arabidopsis plants overexpressing N-terminally masked or truncated proteins revealed that interfering with the function of SAUL1-type proteins resulted in severe growth defects. Our results suggest an ancient origin of ubiquitination at the plasma membrane in the evolution of land plants.

## Introduction

Post-translational modifications of proteins regulate their function and abundance. Ubiquitination of target proteins, in which the C-terminus of ubiquitin is covalently bound to other proteins and attached to itself in growing ubiquitin chains, modifies protein function in case of mono-ubiquitination and triggers degradation of poly-ubiquitinated proteins via the 26S proteasome. E3 ubiquitin ligases are crucial for attachment of ubiquitin to target proteins that are specifically recognized. This specific ubiquitination of proteins via E3 ubiquitin ligases requires the preceding activation of ubiquitin by E1 ubiquitin-activating enzymes and the subsequent transfer to E2 ubiquitin-conjugating enzymes.

Different types of E3 ubiquitin ligases have been classified (Vierstra, 2009). Among these, plant U-box (PUB) proteins represent the most recently identified type of E3 ubiquitin ligases. In Arabidopsis, the group of PUB proteins comprises 64 members (Azevedo et al., 2001; Wiborg et al., 2008). These PUB proteins contain the highly conserved U-box that is required for E2 binding (Pringa et al., 2001). In addition, most of the members contain armadillo (ARM) repeats, which likely constitute interfaces for protein-protein interactions, and are thus named PUB-ARM proteins. They support various functions during plant growth and development such as light and hormone signaling, self-incompatibility, cell death and senescence, as well as stress and pathogen response. Up to now, the latter responses involve the largest number of PUB-ARM proteins indicating a substantial function of this type of E3 ubiquitin ligases for plant survival following stress or pathogen attack (Raab et al., 2009; Yee and Goring, 2009; Mbengue et al., 2010; Bergler and Hoth, 2011; Liu et al., 2011; Park et al., 2011; Salt et al., 2011; Li et al., 2012; Vogelmann et al., 2012).

Specific functions of PUB-ARM proteins depend on their intracellular targeting that is likely defined by the presence of ARM repeat domains. Generally, these domains have been implicated in the localization of eukaryotic ARM repeat proteins to the nucleus, to the cytoplasm, or to polymerized actin (Aberle et al., 1994; Hulsken et al., 1994; Graham et al., 2000; Coates et al., 2006; Yang et al., 2007). For PUB-ARM proteins, localization in nucleus and cytoplasm as well as association with the endoplasmic reticulum (ER) and with the plasma membrane has been demonstrated (Amador et al., 2001; Stone et al., 2003; Cho et al., 2008; Samuel et al., 2008; Drechsel et al., 2011). Plasma membrane-association of the Arabidopsis SAUL1 (SENESCENCE ASSOCIATED UBIQUITIN LIGASE 1) protein depends on an elongated C-terminus with additional ARM repeat domains that are unique to SAUL1 and its two paralogs AtPUB42 and AtPUB43. Whereas deletion of these additional ARM repeat domains resulted in the loss of plasma membrane-association, these C-terminal ARM repeats were sufficient to mediate plasma membrane-association of other cytoplasmic proteins (Drechsel et al., 2011).

Here, we were aiming to identify PUB-ARM proteins with elongated C-terminus carrying additional ARM repeats in other plant species and to test whether the presence of these C-terminal ARM repeat domains also mediates plasma membrane-association of these orthologs of the Arabidopsis SAUL1 protein.

## Materials and methods

### Cloning of DNA constructs

For PCR amplification of DNA fragments, cDNAs were isolated from the aerial parts of young *Arabidopsis thaliana* seedlings grown on soil at long day-conditions (16 h light/8 h dark) at 22°C for 2 weeks, leaves of *Populus trichocarpa* plants grown on soil at long day-conditions (16 h light/8 h dark) for several months, *Physcomitrella patens* grown in sterile culture, and from *Oryza sativa* ssp. *Japonica* cv. *Nipponbare* leaf material from seedlings grown on soil in long day-conditions (16 h light/8 h dark) at 26°C for 4 weeks. Total RNA was isolated with Trizol reagent (Invitrogen, Karlsruhe, Germany). RT-PCRs were performed with the High Capacity RNA-to-cDNA Master Mix (Invitrogen). For generation of fusion proteins between full-length or truncated PUB-ARM proteins and GFP, the respective open reading frames were amplified by PCR from cDNA using the primer pairs listed in Supporting Table S1. The reverse primers harbored a wobble base to generate PCR fragments with or without a stop codon. The amplified fragments were cloned into pENTR/D-TOPO (Invitrogen, Karlsruhe, Germany), verified by sequencing, and recombined into destination vectors pMDC43 (Curtis and Grossniklaus, 2003) for GFP fusion to the N-terminus and pK7FWG2.0 (Karimi et al., 2002) for fusion of GFP to the C-terminus.

### Protoplast isolation, transformation, and confocal analysis

Protoplasts were isolated from fully expanded leaves of 3–4 week-old Arabidopsis plants grown on soil. Leaves were roughened using sandpaper, transferred to protoplasting buffer (500 mM sorbitol, 1 mM CaCl_2_, 0.03% pectolyase Y23, 0.75% cellulose YC and 10 mM MES-KOH, pH 5.6–6.0), and incubated in the dark at 22°C for 1.5 h with gentle agitation (60–75 rpm). Protoplasts were separated from undigested material by filtration through a 50 μm nylon mesh and sedimented by centrifugation for 8 min at 100 × g. The pellet was resuspended in MaMg buffer (400 mM sorbitol, 15 mM MgCl_2_, 5 mM MES-KOH, pH 5.6). Protoplast transformation was essentially performed as previously described (Abel and Theologis, 1994). Transformed protoplasts were transferred into small petri dishes and incubated for 24 h in the dark at 22°C prior to analysis by confocal laser scanning microscopy as described previously (Drechsel et al., 2011).

### Transient transformation of tobacco leaves

For transient transformation of *Nicotiana benthamiana* leaves, *Agrobacterium tumefaciens* strain C58C1 (Deblaere et al., 1985) harboring the respective DNA construct was grown at 29°C in LB supplemented with 50 μg ml^−1^ kanamycin to the stationary phase. Bacteria were sedimented by centrifugation at 5000 g for 15 min at room temperature and resuspended in infiltration buffer (10 mM MgCl_2_, 10 mM MES, KOH pH5.7). Cells were infiltrated into the abaxial air spaces of 2–4-week-old *N. benthamiana* plants. GFP fluorescence was monitored by confocal laser scanning microscopy 24–48 h past infiltration as described previously (Drechsel et al., 2011).

### Phylogenetic analysis

A multiple sequence alignment of 150 PUB-ARM proteins from Arabidopsis (*A. thaliana)*, rice (*Oryza sativa*), poplar (*Populus trichocarpa*) and moss (*Physcomitrella patens*) was generated employing MAFFT 6 (Katoh et al., 2005) with the E-INS-i routine and the BLOSUM 45 matrix at http://mafft.cbrc.jp/alignment/server/. In the first phylogenetic analysis, the full alignment (2683 amino acid positions) was used, in a second approach a reduced alignment without any gap position (210 amino acids positions) was employed. Bayesian phylogenetic analyses were performed with MrBayes 3.1.2 (Huelsenbeck and Ronquist, 2001). We assumed the WAG model of amino acid evolution (Whelan and Goldman, 2001) with gamma distribution of substitution rates. Metropolis-coupled Markov chain Monte Carlo (MCMCMC) sampling was performed with one cold and three heated chains. Two independent runs were performed in parallel for 5 million generations each. Starting trees were random and the trees were sampled every 1000th generation. Posterior probabilities were estimated on the final 4000 trees (burnin = 1000).

## Results

### Plasma membrane-association of PUB43 depends on its C-terminal ARM repeat domain

**Figure 1:**
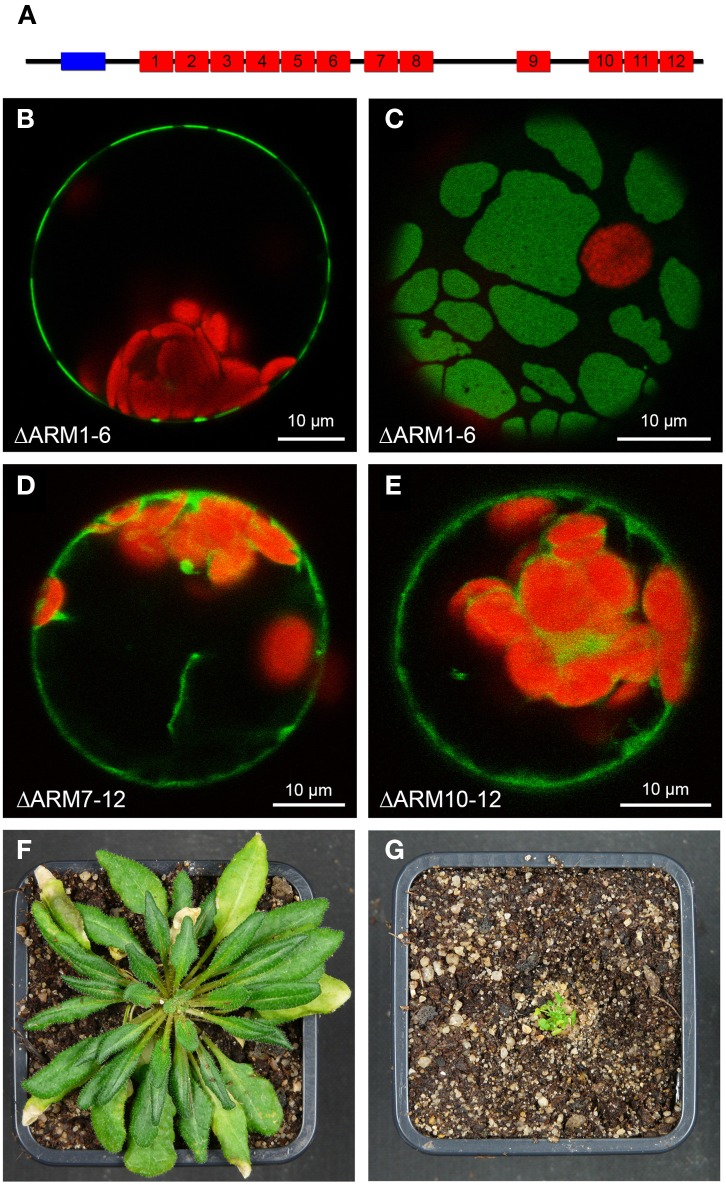
**Involvement of C-terminal ARM repeats in plasma membrane-association of AtPUB43. (A)** Schematic representation of the AtPUB43 protein domain structure. U-box and ARM repeats are depicted in blue and red, respectively. **(B)** Confocal analysis of fluorescence of an Arabidopsis leaf protoplast expressing AtPUB43ΔARM_1−6_-GFP fusion proteins. GFP fluorescence depicted in green was only detected at the plasma membrane. Chlorophyll auto-fluorescence within the chloroplasts is depicted in red. **(C)** Confocal analysis of fluorescence of an Arabidopsis leaf protoplast expressing AtPUB43ΔARM_1−6_-GFP fusion proteins. The representative top-view showed that GFP signals and thus the AtPUB43ΔARM_1−6_-GFP fusion proteins were localized in membrane patches. **(D)** Confocal analysis of fluorescence of an Arabidopsis leaf protoplast expressing AtPUB43ΔARM_7−12_-GFP fusion proteins. GFP fluorescence depicted in green was detected in the cytosol. Chlorophyll auto-fluorescence within the chloroplasts is depicted in red. **(E)** Confocal analysis of fluorescence of an Arabidopsis leaf protoplast expressing AtPUB43ΔARM_10−12_-GFP fusion proteins. GFP fluorescence depicted in green was detected in the cytosol. Chlorophyll auto-fluorescence within the chloroplasts is depicted in red. **(F)** Growth phenotype of wildtype plants grown for 12 weeks in short-day conditions. Photon flux density was 80–100 μmol m^−2^ s^−1^. **(G)** Growth phenotype of CaMV35S::YFP-SAUL1 plants grown for 12 weeks in short-day conditions. Photon flux density was 80-100 μmol m^−2^ s^−1^.

**Figure 2:**
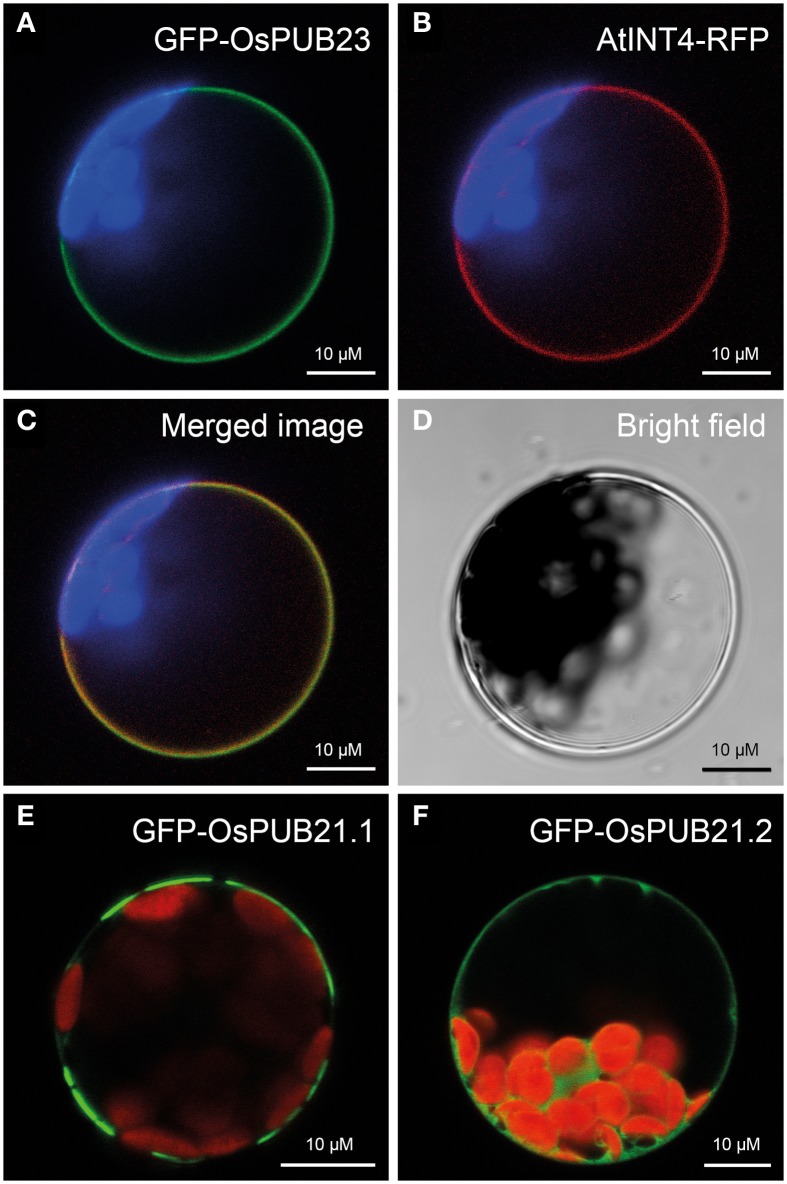
**Localization of rice SAUL-type PUB-ARM proteins at the plasma membrane. (A)** Localization of GFP-OsPUB23 fusion proteins at the plasma membrane of Arabidopsis protoplasts. Confocal laser scanning microscopy detected fluorescence of GFP-OsPUB23 proteins depicted in green at the plasma membrane of protoplasts co-expressing GFP-OsPUB23 and AtINT4-RFP. Chlorophyll auto-fluorescence within the chloroplasts is depicted in blue. **(B)** Localization of AtINT4-RFP fusion proteins at the plasma membrane. Confocal laser scanning microscopy detected fluorescence of AtINT4-RFP proteins depicted in red at the plasma membrane of protoplasts co-expressing GFP-OsPUB23 and AtINT4-RFP. Chlorophyll auto-fluorescence within the chloroplasts is depicted in blue. **(C)** Merged image of **(A,B)**. Yellow signals derived from the overlap of green and red fluorescences from **(A,B)**. **(D)** Bright filed image of the protoplast analyzed in **(A–C)**. **(E)** Localization of GFP-OsPUB21.1 fusion proteins at the plasma membrane. Confocal laser scanning microscopy detected fluorescence of GFP-OsPUB21.1 proteins depicted in green at the plasma membrane of transformed protoplasts. Chlorophyll auto-fluorescence within the chloroplasts is depicted in red. **(F)** Localization of GFP-OsPUB21.2 fusion proteins at the plasma membrane. Confocal laser scanning microscopy detected fluorescence of GFP-OsPUB21.1 proteins depicted in green in the cytosol of transformed Arabidopsis protoplasts. Chlorophyll auto-fluorescence within the chloroplasts is depicted in red.

Plasma membrane-association was observed for the two SAUL1 paralogs AtPUB42 and AtPUB43, but not for any other member of the Arabidopsis PUB-ARM protein family (Drechsel et al., 2011). For SAUL1 it has been demonstrated that this specific localization depends on the additional ARM repeat domain in the C-terminus that is unique to SAUL1, AtPUB42, and AtPUB43. The domain structure of AtPUB43 is schematically depicted in Figure 1A. To test whether the C-terminal ARM repeat domain is also essential for plasma membrane-association of AtPUB43, we analyzed the localization of fusion proteins between GFP and truncated AtPUB43 proteins by confocal laser scanning microscopy on transformed Arabidopsis protoplasts. As for SAUL1, deletion of the N-terminal part of AtPUB43 in AtPUB43ΔARM_1−6_-GFP proteins did not result in the loss of plasma membrane-association (Figure 1B). This deletion protein, however, was not equally distributed in the plasma membrane, but occurred in large patches (Figures 1B,C). A similar pattern has been observed for SAUL1 proteins with truncated and/or masked N-terminus previously (Drechsel et al., 2011). In a next step, either ARM repeats 7–12 or 10–12 were deleted in AtPUB43. Both proteins, AtPUB43ΔARM_7−12_-GFP and AtPUB43ΔARM_10−12_-GFP, were localized to the cytoplasm and not to the plasma membrane (Figures 1D,E). These data indicated that indeed the C-terminal ARM repeat domain is essential and sufficient for plasma membrane-association of AtPUB43 and that this is a general feature of SAUL1-type E3 ubiquitin ligases. The analysis of transgenic Arabidopsis plants overexpressing GFP-AtPUB43ΔARM_1−6_ showed that the C-terminus by itself had no effect on plant growth and development (Figure S1). To test whether masking the N-terminus of SAUL1-type proteins, which eventually leads to patchy distribution of the protein at the plasma membrane (Drechsel et al., 2011 and Figure 2E), may affect growth and development, we analyzed CaMV35S::YFP-SAUL1 plants. Indeed, overexpression of YFP-SAUL1 fusion proteins resulted in a severe growth defect when compared to growth of wildtype plants (Figures 1F,G).

### SAUL1-type PUB-ARM proteins from rice are associated to the plasma membrane

Recently, the PUB-ARM protein family has been described in rice (Zeng et al., 2008). Five members of this protein family, namely OsPUB21, OsPUB22, OsPUB23, OsPUB24, and OsPUB25 possess an elongated C-terminus and carry an additional C-terminal ARM repeat domain like their Arabidopsis orthologs. We investigated whether the rice SAUL1 orthologs were also associated to the plasma membrane. For that purpose, GFP-OsPUB23 fusion proteins were expressed in Arabidopsis protoplasts and their fluorescence analyzed by confocal laser scanning microscopy. GFP fluorescence indicated plasma membrane-association of GFP-OsPUB23 (Figure 2A). To confirm this localization, GFP-OsPUB23 was co-expressed with AtINT4-RFP. The AtINT4 inositol transport protein was localized to the plasma membrane as described previously (Figure 2B, c.f. Schneider et al., 2006). Merging green and red fluorescence of GFP-OsPUB23 and AtINT4-RFP, respectively, clearly showed that OsPUB23 is associated to the plasma membrane (Figures 2C,D). When isolating the cDNA clone of *OsPUB21*, we not only found the long form *OsPUB21.1*, but also recovered the truncated *OsPUB21.2* missing the additional C-terminal ARM repeat domain. Whereas GFP-OsPUB21.1 was also associated to the plasma membrane (Figure 2E), GFP-OsPUB21.2 was not localized to the plasma membrane but to the cytoplasm (Figure 2F). Masking the N-terminus of Os-PUB21.1 by GFP again resulted in patchy distribution at the plasma membrane (Figure 2E). Indeed, rice SAUL1-type PUB-ARM proteins were associated to the plasma membrane and this localization was dependent on the elongated C-terminus carrying the additional ARM repeat domain.

### Identification and phylogenetic analyses of PUB-ARM proteins from *populus trichocarpa* and *physcomitrella patens*

To test whether SAUL1-type PUB-ARM proteins with additional C-terminal ARM-repeats exist in other plants and in mosses, we were aiming to identify PUB-ARM proteins in *Populus trichocarpa* and in the moss *Physcomitrella patens*. For that purpose, we used the protein sequences of SAUL1, AtPUB42, and AtPUB43 including their elongated C-terminus to BLAST search the respective genomes on the phytozome website (www.phytozome.net). When using this approach to identify PUB-ARM proteins in rice, we could re-identify all PUB-ARM proteins that were recently published by Zeng et al. (2008). We therefore decided to follow this approach on *P. trichocarpa* and *P. patens*. In *P. trichocarpa* and *P. patens*, 50 and 31 PUB-ARM proteins were identified, respectively.

**Figure 3:**
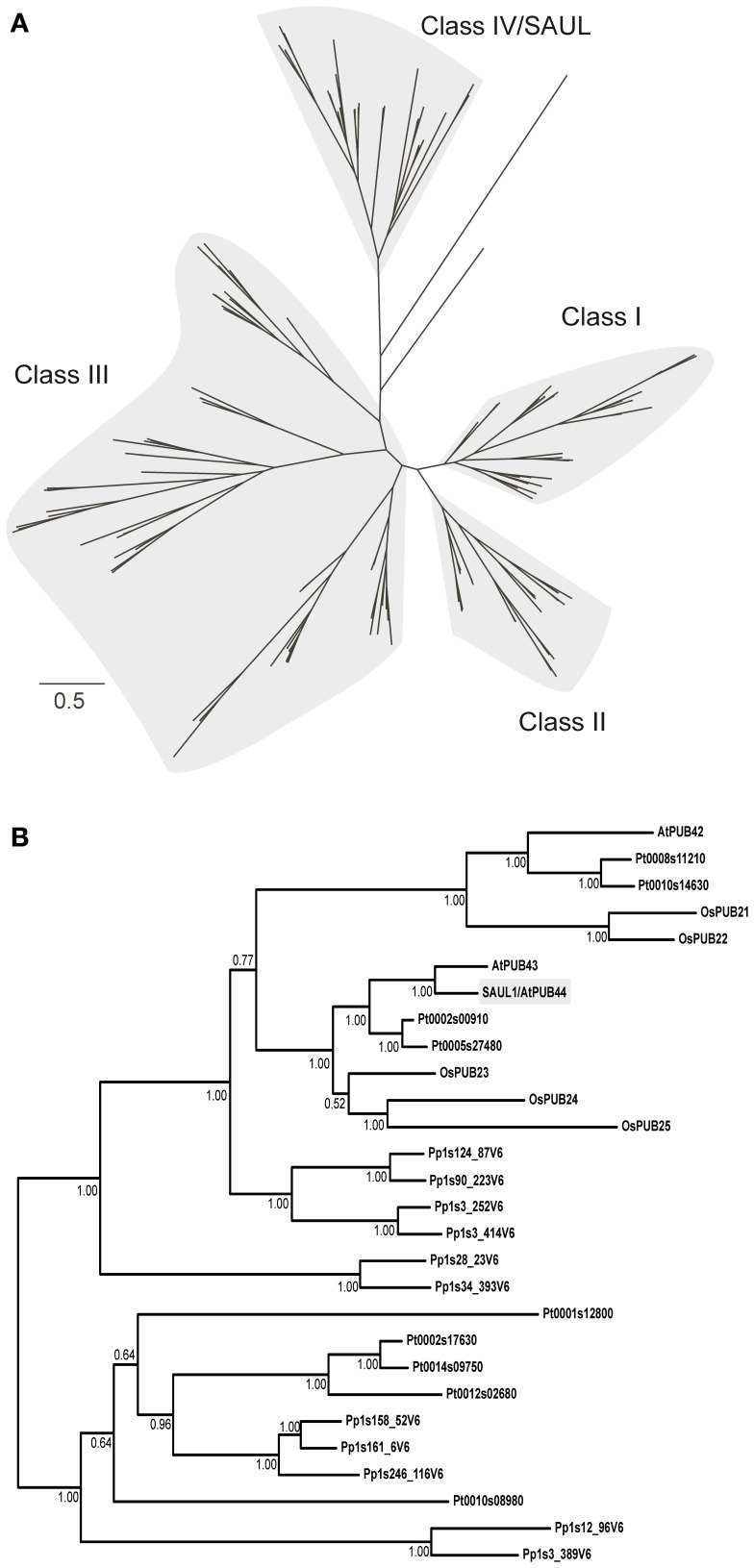
**Phylogenetic relationship of PUB-ARM proteins of Arabidopsis, rice, poplar and moss. (A)** Simplified Bayesian phylogenetic tree depicting the relationship among class I–IV PUB-ARM proteins, as defined by Zeng et al. (2008). The bar represents 0.5 PAM distance. The tree derived from the analysis of the 2683 amino acid alignment; the full tree in Supporting Figure S2. **(B)** Subtree of **(A)** depicting the relationship among class IV proteins, which include SAUL1-type proteins. Arabidopsis SAUL1 (AtPUB44) is highlighted in gray; the numbers at the nodes are Bayesian posterior probabilities.

Two multiple sequence alignments were applied for Bayesian phylogenetic analyses. The first alignment covered 2683 amino acid positions and included the full-length proteins. In the second alignment all gaps were removed, resulting in 210 positions. Both alignments gave very similar trees, which recovered the classes I–IV of PUB-ARM proteins, as defined by Zeng et al. (2008) (Figure 3A; Supporting Figures S2, S3). SAUL1 and SAUL-like paralogs of Arabidopsis and rice are members of class IV. SAUL1 (AtPUB44) and AtPUB43 are closely related and form a common branch with three PUB-ARM proteins from rice (OsPUB23–25) and two from poplar (Pt0002s00910 and Pt0005s27480) (Figure 3B). AtPUB42 is on a different branch within class IV and is related to rice OsPUB21 and 22, as well as two proteins of poplar (Pt0008s11210 and Pt0010s14630). Six proteins of the bryophyte *P. patens* are related to the clade of SAUL1-like proteins of the vascular plants. In addition, five proteins from *P. trichocarpa* and five from *P. patens* form a distinct clade within class IV, which is more distantly related to SAUL1. SAUL1 orthologs within the class IV proteins displayed sequences identities of more than 40%. Comparison of SAUL1 with class I–III PUB-ARM proteins revealed low identity scores of less than 26%. In all cases and in contrast to all other PUB-ARM proteins, SAUL1-type PUB-ARM proteins consist of a clearly higher number of amino acids due to their specific domain organization, namely their elongated C-terminus that contains additional ARM repeat domains. Based on these criteria, additional BLAST searches identified putative SAUL1-type PUB-ARM proteins in all land plants listed on the phytozome website, suggesting conservation in land plants (not shown).

### SAUL1-type PUB-ARM proteins from *populus trichocarpa* and *physcomitrella* patens are associated with the plasma membrane

**Figure 4:**
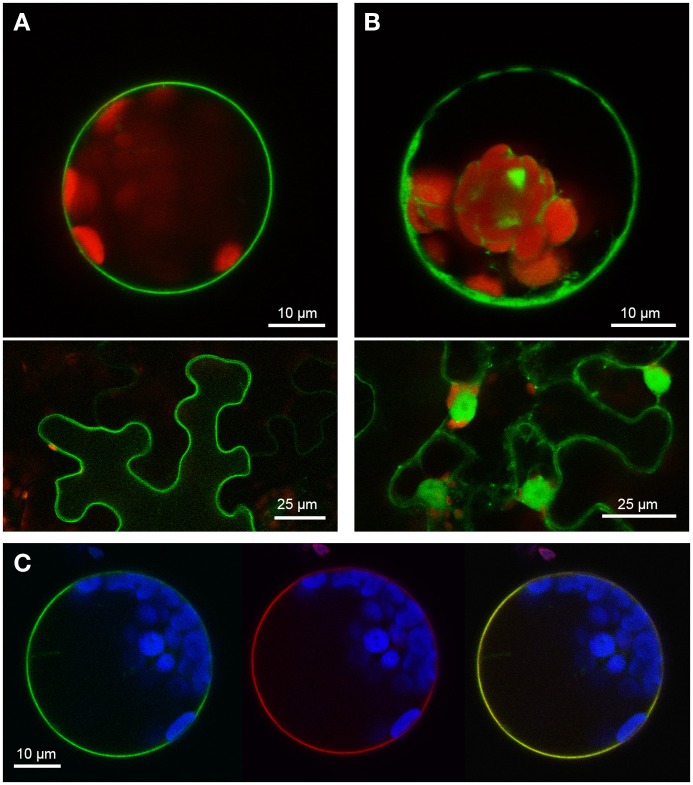
**Localization of SAUL-type PUB-ARM proteins from *Populus trichocarpa* at the plasma membrane. (A)** Confocal analysis of fluorescence of Arabidopsis leaf protoplasts (top panel) and tobacco leaf epidermal cells (bottom panel) expressing GFP-Pt0005s27480 fusion proteins. GFP fluorescence depicted in green was detected at the plasma membrane in both cell types. Chlorophyll auto-fluorescence within the chloroplasts is depicted in red. **(B)** Confocal analysis of fluorescence of Arabidopsis leaf protoplasts (top panel) and tobacco leaf epidermal cells (bottom panel) expressing GFP-Pt0004s02840 fusion proteins. GFP fluorescence depicted in green was detected in cytosol and nucleus in both cell types. Chlorophyll auto-fluorescence within the chloroplasts is depicted in red. **(C)** Localization of GFP-Pt0005s27480 fusion proteins at the plasma membrane of Arabidopsis protoplasts. Confocal laser scanning microscopy detected fluorescence of GFP-Pt0005s27480 proteins depicted in green (left) and RFP-UmSrt1 (middle) proteins depicted in red at the plasma membrane of protoplasts co-expressing GFP-Pt0005s27480 and RFP-UmSrt1. Merging both images resulted in yellow signals (right) derived from the overlap of green (GFP-Pt0005s27480) and red (RFP-UmSrt1) fluorescences. Chlorophyll auto-fluorescence within the chloroplasts is depicted in blue.

**Figure 5:**
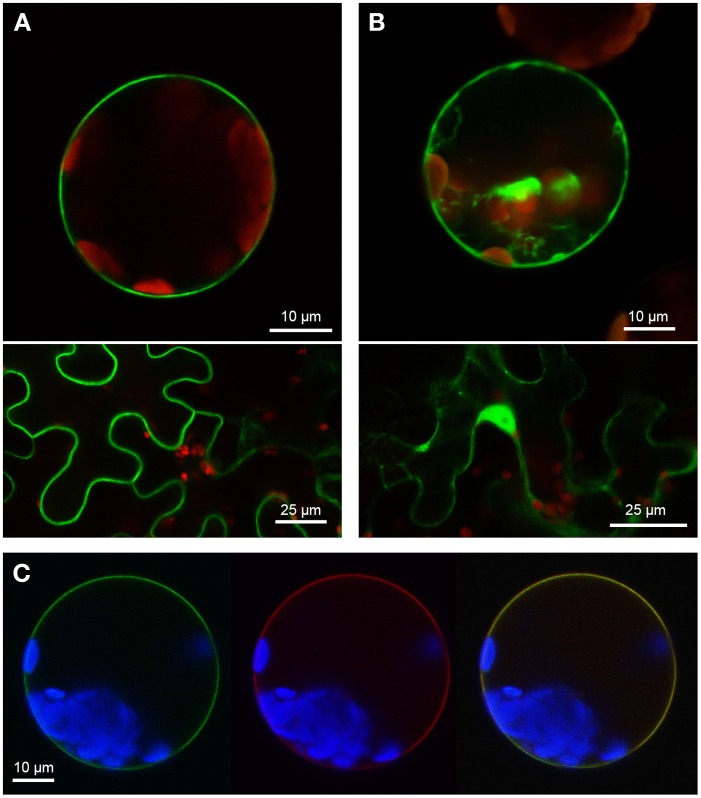
**Localization of SAUL-type PUB-ARM proteins from *Physcomitrella patens* at the plasma membrane. (A)** Confocal analysis of fluorescence of Arabidopsis leaf protoplasts (top panel) and tobacco leaf epidermal cells (bottom panel) expressing GFP-Pp1s3_414V6 fusion proteins. GFP fluorescence depicted in green was detected at the plasma membrane in both cell types. Chlorophyll auto-fluorescence within the chloroplasts is depicted in red. **(B)** Confocal analysis of fluorescence of Arabidopsis leaf protoplasts (top panel) and tobacco leaf epidermal cells (bottom panel) expressing GFP-Pp1s67_203V6 fusion proteins. GFP fluorescence depicted in green was detected in cytosol and nucleus in both cell types. Chlorophyll auto-fluorescence within the chloroplasts is depicted in red. **(C)** Localization of GFP-Pp1s3_414V6 fusion proteins at the plasma membrane of Arabidopsis protoplasts. Confocal laser scanning microscopy detected fluorescence of GFP-Pp1s3_414V6 proteins depicted in green (left) and RFP-UmSrt1 (middle) proteins depicted in red at the plasma membrane of protoplasts co-expressing GFP-Pp1s3_414V6 and RFP-UmSrt1. Merging both images resulted in yellow signals (right) derived from the overlap of green (GFP-Pp1s3_414V6) and red (RFP-UmSrt1) fluorescences. Chlorophyll auto-fluorescence within the chloroplasts is depicted in blue.

Two PUB-ARM proteins from each organism, one SAUL1-type and one without the additional C-terminal ARM repeat domain, were selected to investigate conservation of SAUL1-type plasma membrane-association in *Populus trichocarpa* and *Physcomitrella patens*. Confocal laser scanning microscopy on transformed Arabidopsis leaf cell protoplasts and tobacco leaf epidermal cells analyzed localization of GFP fusion proteins. The poplar SAUL1-type PUB-ARM protein Pt0005s27480 (POPTR_0005s27480) localized to the plasma membrane as indicated by GFP fluorescence of GFP-Pt0005s27480 (Figure 4A) or Pt0005s27480-GFP (not shown) fusion proteins. In contrast, the same approach showed that the poplar PUB-ARM protein Pt0004s02840 (POPTR_0004s02840) that is lacking the C-terminal ARM repeat domain was localized to cytoplasm and nucleus (Figure 4B and not shown). The same results were obtained in *Physcomitrella patens*. Whereas fusion proteins with the SAUL1-type PUB-ARM protein Pp1s3_414V6 were associated with the plasma membrane, the Pp1s67_203V6 protein that also does not contain the additional ARM repeat domain localized to cytoplasm and nucleus (Figures 5A,B). To confirm localization of Pt0005s27480 and Pp1s3_414V6 at the plasma membrane, both GFP fusion proteins were co-expressed with the plasma membrane sugar transport protein UmSrt1 from the phytopathogenic fungus *Ustilago maydis* fused to RFP (Figures 4C, 5C). The UmSrt1 transport protein was localized to the plasma membrane as described previously (c.f. Drechsel et al., 2011). Merging green and red fluorescence of GFP-Pt0005s27480 and RFP-UmSrt1 (Figure 4C) or GFP-Pp1s3_414V6 and RFP-UmSrt1 (Figure 5C) resulted in yellow signals and clearly showed that both SAUL1-type proteins are associated to the plasma membrane.

## Discussion

Plant growth and development depend on specific degradation of proteins or on modification of protein functions following the attachment of ubiquitin. PUB armadillo repeat (PUB-ARM) proteins represent one class of E3 ubiquitin ligases that function in ubiquitination of target proteins. Whereas the majority of Arabidopsis PUB-ARM proteins were localized to the cytoplasm and/or nucleus, SAUL1 and its paralogs AtPUB42 and AtPUB43 were associated with the plasma membrane (Drechsel et al., 2011). This unique association with the plasma membrane is due to their exceptional protein structure. Alike all other PUB-ARM proteins, SAUL1-type proteins contain the U-box at the N-terminus important for E2 binding and a neighboring set of ARM repeats that is likely crucial for target binding. However, in addition to these domains SAUL1-type PUB-ARM proteins are equipped with an additional set of ARM repeats at the elongated C-terminus. This domain has been shown to be essential and sufficient for plasma membrane-association of Arabidopsis SAUL1-type PUB-ARM proteins (Figure 1, Drechsel et al., 2011).

Here, we showed that rice SAUL1 orthologs, which have been identified by Zeng et al. (2008), also localized to the plasma membrane, and that the additional ARM repeat domain at the C-terminus is essential for this localization (Figure 2). To further prove that additional ARM repeats in an elongated C-terminus and thus plasma membrane-association are conserved features of SAUL1-type PUB-ARM proteins, we identified PUB-ARM proteins in *P. trichocarpa* and *P. patens.* In both plant species our search identified a group of SAUL1-type proteins, and we could demonstrate that these were associated to the plasma membrane indicating that this was a conserved function (Figures 4, 5). Our phylogenetic analyses identified SAUL1-type proteins as members of class IV PUB-ARM proteins, as defined by Zeng et al. (2008) (Figure 3). The basic topology of the tree of PUB-ARM proteins followed that outlined by Zeng et al. (2008) and recovered the classes I, II, and IV identified in rice. Only class III was found paraphyletic in our analyses, comprising of in fact three distinct clades. In addition to Arabidopsis and rice, SAUL1-like proteins were also found in poplar and moss, suggesting an ancient origin of this type of ubiquitin ligase early in the evolution of land plants. There is a second group of proteins within class IV that is related to the SAUL1-like proteins, which has no orthologs in Arabidopsis and rice (Figure 3B). It remains to be demonstrated whether these proteins are also associated with the membrane and have functions similar to SAUL1.

To unravel and understand the function of PUB-ARM proteins, their target proteins have to be identified. To this end, we could show that interfering with the function of SAUl1-type proteins by masking the N-terminus with YFP resulted in severe growth defects in CaMV35S::YFP-SAUL1 plants (Figure 1). The N-terminal YFP may lead to conformational changes in SAUL1 and thus prevent correct binding to the respective E2 enzyme through the U-box that is contained in the SAUL1 N-terminus (Aravind and Koonin, 2000; Pringa et al., 2001). The future identification of SAUL1 target proteins will show whether masking the N-terminus may affect target recognition.

For some cytosolic/nuclear PUB-ARM proteins, the substrates have been identified. In the proteasome, subunits of the 19S regulatory particle represent targets of CaPUB1 and AtPUB22 (Cho et al., 2006, 2008). Degradation of these subunits results in reduced levels of functional 26S proteasomes, which may affect environmental stress responses. In rice, the PUB-ARM E3 ligase SPL11 (SPOTTED LEAF11) monoubiquitinates and thus negatively regulates SPIN1 (SPL11-interacting protein1), a member of the STAR (Signal Transduction and Activation of RNA) protein family. SPIN1 functions as a negative regulator of flowering in rice (Vega-Sanchez et al., 2008). The Arabidopsis PUB-ARM proteins AtPUB12 and AtPUB13 poly-ubiquitinate FLS2 (PRR FLAGELLIN-SENSING 2), thereby promote its degradation, and have a function in flagellin-induced immune responses (Lu et al., 2011). Recently, it was demonstrated that AtPUB22 mediated degradation of components of the exocyst complex thus affecting PAMP-triggered signaling (Stegmann et al., 2012). In contrast to the described examples, target proteins of plasma membrane-associated PUB-ARM proteins have not been identified yet.

A possible function of ubiquitination at the plasma membrane mediated by SAUL1-type PUB armadillo repeat E3 ubiquitin ligases could be the internalization of membrane proteins and thus the regulation of plasma membrane protein composition. The requirement of ubiquitin as a signal for internalization has primarily been demonstrated in yeast and mammals (Rotin et al., 2000; Hicke and Dunn, 2003). In plants, endocytosis can also remove transporters (PIN1) and receptors (BRI1, FLS2) from the plasma membrane (Geldner et al., 2001; Russinova et al., 2004; Robatzek et al., 2006). Generally, endosomal removal cannot only lead to receptor inactivation and down-regulation of signaling, but may also stimulate signaling in case of accumulation of activated receptors in endosomes. Ubiquitination is involved in endocytosis of the Arabidopsis flagellin receptor FLS2 (FLAGELLIN-SENSING2) that results in its degradation (Gohre et al., 2008). Recently, it has been shown that flagellin induces association of FLS2 with the PUB-ARM proteins AtPUB12 and AtPUB13, which direct ubiquitination and turnover of FLS2 (Lu et al., 2011). The down-regulation of the plasma membrane transport proteins IRT1 (IRON-REGULATED TRANSPORTER1) and BOR1 (BORON TRANSPORTER1) also requires ubiquitination (Barberon et al., 2011; Kasai et al., 2011). Endocytosis and changes in protein stability were also induced by artificial mono-ubiquitination of the PM ATPase PMA and PIN2 (Herberth et al., 2012; Leitner et al., 2012). In addition, these and other studies link ubiquitination to the endosomal sorting complex required for transport (ESCRT) (Raiborg and Stenmark, 2009; Furlan et al., 2012; Scheuring et al., 2012). We hypothesize that plasma membrane-associated SAUL1-type E3 ubiquitin ligases may be responsible for mono-ubiquitination of membrane proteins to induce their endocytosis. Alternatively, they could be involved in processing plasma membrane-anchored regulatory proteins via poly-ubiquitination (Hoppe et al., 2001). Future identification of targets of SAUL1-type E3 ubiquitin ligases will help to unravel their molecular function at the plasma membrane.

### Conflict of interest statement

The authors declare that the research was conducted in the absence of any commercial or financial relationships that could be construed as a potential conflict of interest.

## Supplementary material

Figure S1 **Growth and development of Arabidopsis plants overexpressing GFP-AtPUB43ΔARM_1–6_ fusion proteins**.

Figure S2 **Phylogeny of PUB-ARM proteins of Arabidopsis, rice, poplar, and moss, as recovered by Bayesian analysis of the alignment covering 150 Proteins and 2683 amino acid positions**.

Figure S3 **Phylogeny of PUB-ARM proteins of Arabidopsis, rice, poplar, and moss, as recovered by Bayesian analysis of the alignment covering 150 Proteins and 210 amino acid positions**.

Table S1 **Primer pairs for amplification the respective open reading frames by PCR**.
