# Supplementary material for: Plasma membrane-association of SAUL1-type plant U-box armadillo repeat proteins is conserved in land plants
Source: Front Plant Sci. 2014 Feb 19;5:37. doi: 10.3389/fpls.2014.00037 (PMC3928556; doi:10.3389/fpls.2014.00037)
Supplement: Figure S1 — Growth and development of Arabidopsis plants overexpressing GFP-AtPUB43ΔARM1–6 fusion proteins. [file DataSheet1.PDF]

Supplementary Table S1

Fragment	Primer #1	Primer #2
PUB43ΔARM$_{1\text{-}6}$	CACCATGCCTGTTGGACCTCATCAC	GGATCMACCAATGTTGGTGAATATAC
PUB43ΔARM$_{7\text{-}12}$	CACCATGGCTGGAAGTGGAAGTTG	GGATCMAACTTTGTCGAAGTCGTAC
PUB43ΔARM$_{10\text{-}12}$	CACCATGGCTGGAAGTGGAAGTTG	GGATCMGCTCAGACATGAAAAGATG
OsPUB23	CACCATGGCAGAAGGTCAGG	GGATCMTGCTGCCCCCATC
OsPUB21	CACCATGTTGCTGGTAGTCAGTGG	GGATCMGAATTCCACGGACATG
Pt0005s27480	CACCATGGCTGAAAGCTGGGATGG	GGATCMGAAAGCGGTCTGACCTGTATT
Pt0004s02840	CACCATGGCTACACACATCCCTGA	GGATCMCCCCCTACAACTTAAATTTTGC
Pp1s3_3414V6	CACCATGGATGCGATTAAACGTGACG	GGATCMCCGTCTCCTAATCATGGG
Pp1s6_67203V6	CACCATGGCGCTTGGCTCTAGC	TTACACTAGATCGGTCTTAAATCGC

Supplementary Figure S1

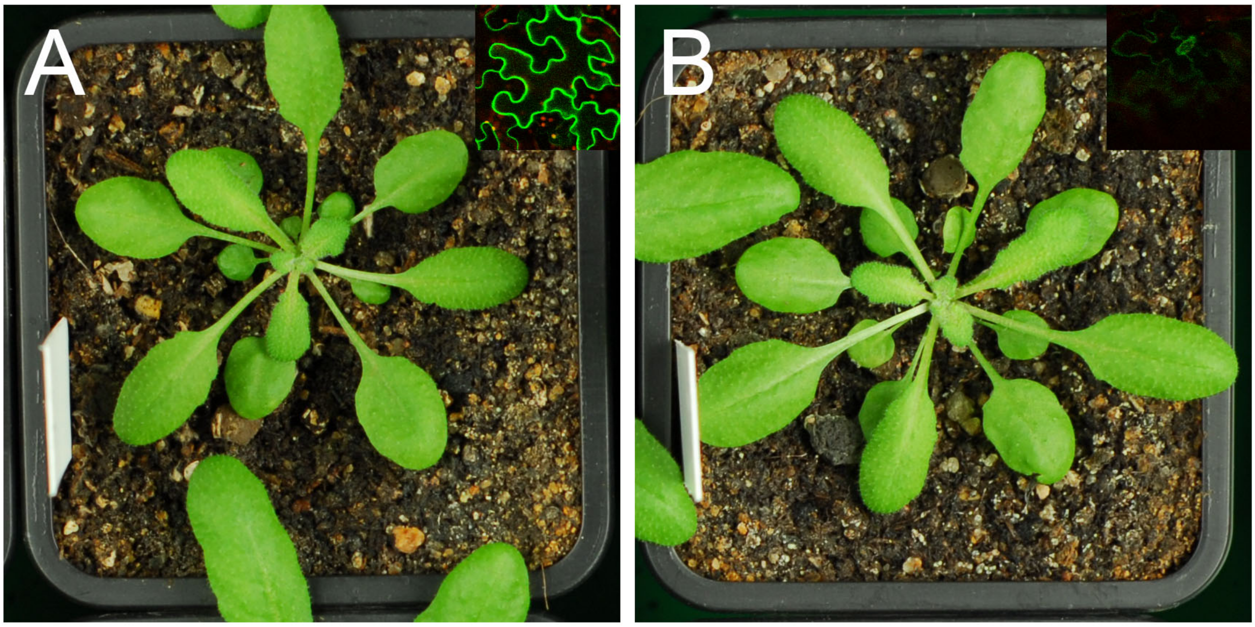

Supplementary Figure S2

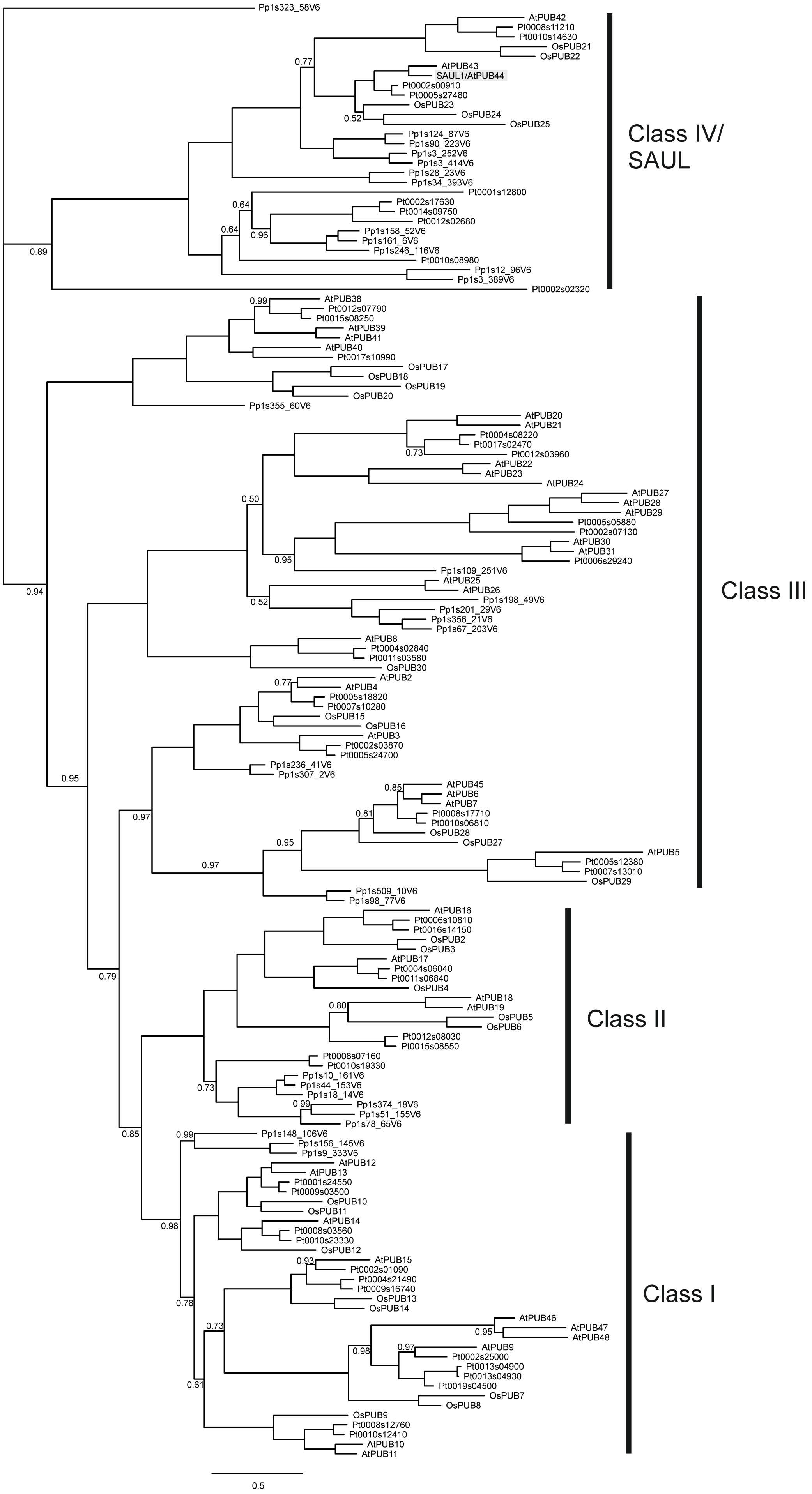

Supplementary Figure S3

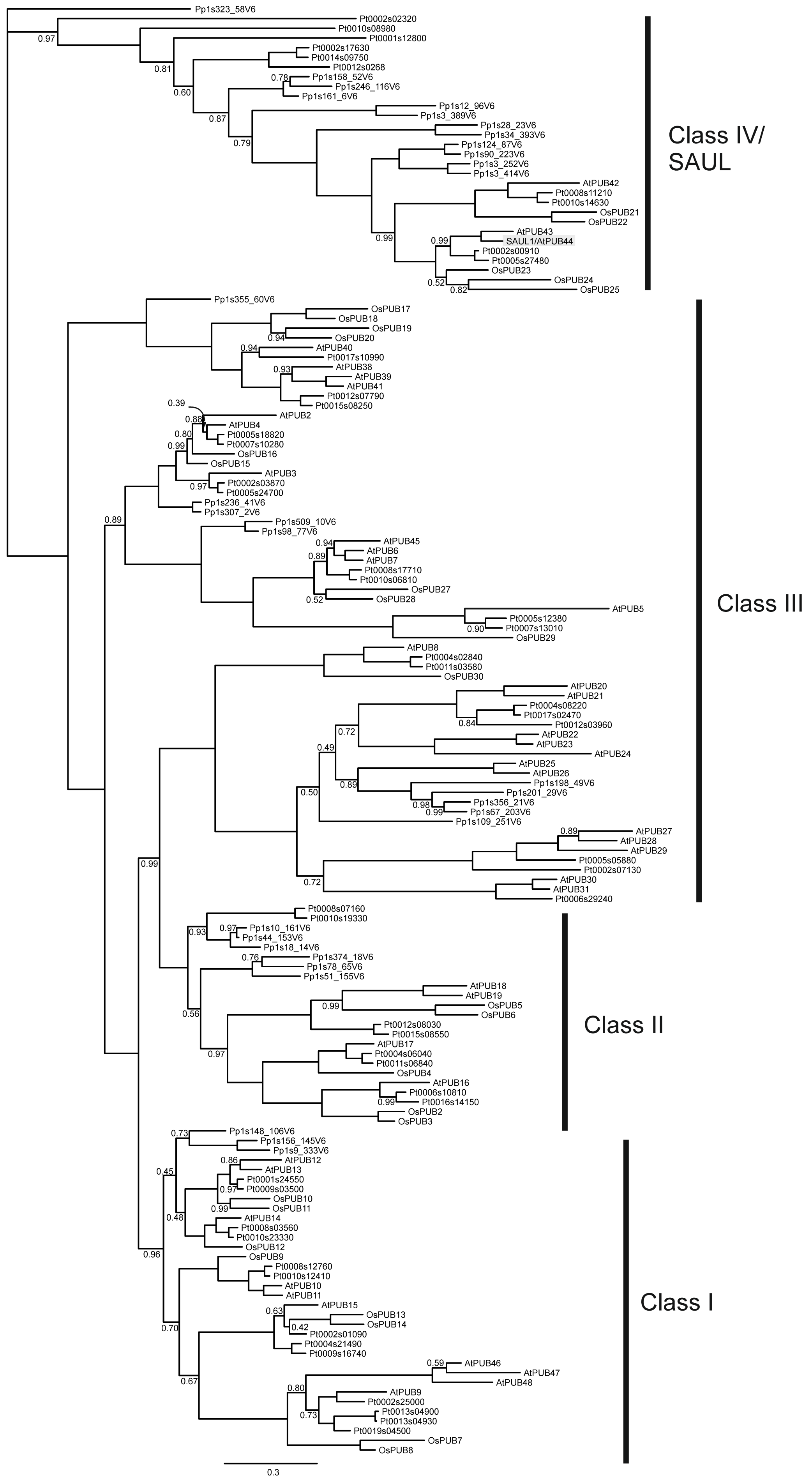

Legends to the Supplementary Figures

Supplementary Figure S1. Indistinguishable growth and developmental phenotypes of two independent lines of transgenic GFP-AtPUB43ΔARM$_{1-6}$ plants expressing different amounts of the fusion protein between GFP and AtPUB43ΔARM$_{1-6}$. (**A**) Phenotype of a transgenic plant expressing high amounts of GFP-AtPUB43ΔARM$_{1-6}$ as indicated by GFP fluorescence (inset). (**B**) Phenotype of a transgenic plant expressing low amounts of GFP-AtPUB43ΔARM$_{1-6}$ as indicated by GFP fluorescence (inset).

Supplementary Figure S2. Phylogeny of PUB-ARM proteins of Arabidopsis, rice, poplar and moss, as recovered by Bayesian analysis of the alignment covering 150 proteins and 2,683 amino acid positions. The tree was rooted with Pp1s323_58V6 for visualisation purposes only. Arabidopsis SAUL1 (AtPUB44) is highlighted in grey, classes I – IV of PUB-ARM proteins as defined by Zeng et al. (2008) are indicated. The numbers at the nodes are posterior probabilities; nodes without number received maximum Bayesian support (1.0 posterior probability). The bar represents 0.5 PAM distance.

Supplementary Figure S3. Phylogeny of PUB-ARM proteins of Arabidopsis, rice, poplar and moss, as recovered by Bayesian analysis of the alignment covering 150 proteins and 210 amino acid positions. The tree was rooted with Pp1s323_58V6 for visualisation purposes only. Arabidopsis SAUL1 (AtPUB44) is highlighted in grey, classes I – IV of PUB-ARM proteins as defined by Zeng et al. (2008) are indicated. The numbers at the nodes are posterior probabilities; nodes without number received maximum Bayesian support (1.0 posterior probability). The bar represents 0.3 PAM distance.
